# Quasi-Static and Dynamic Interfacial Bond Performance Between Ultra-Early-Strength Unsaturated Polyester Polymer Concrete and Ordinary Portland Concrete Under Flexural Loadings

**DOI:** 10.3390/polym18172144

**Published:** 2026-09-02

**Authors:** Wanhui Feng, Ruihui Lu, Jingxuan Ma, Yi Zhou, Jinfeng Xu, Yuxuan Guan, Shizhe Chen, Song Li

**Affiliations:** College of Urban and Rural Construction, Zhongkai University of Agriculture and Engineering, Guangzhou 510225, China; whfeng@zhku.edu.cn (W.F.);

**Keywords:** unsaturated polyester resin concrete, bonding strength, repair materials, flexural properties, strain-rate effect

## Abstract

Unsaturated polyester polymer concrete (UPPC) is a promising rapid repair material for pavement engineering, while the age-dependent and rate-dependent bonding behaviors between UPPC and ordinary Portland concrete (OPC) substrate remains poorly understood, restricting its application in heavy-traffic scenarios. This study systematically investigates the quasi-static and dynamic bond performance of the UPPC-OPC interface at four curing ages (1 d, 3 d, 7 d, 28 d) under flexural loads. Quasi-static three-point bending tests show that the 28 d interfacial bond strength reaches 4.62 MPa, 97% higher than that of the OPC-OPC control group. Dynamic flexural tests via a 100 mm diameter split Hopkinson pressure bar reveal the strain-rate effect of the dynamic increase factor (DIF). DIF decreases below 1.0 at low strain rates (<10 s^−1^) due to weakened viscoelastic stress relaxation of partially cured unsaturated polyester resin, while it rises above 1.0 at higher strain rates induced by polymer hardening and inertial confinement. Combined with scanning electron microscopy characterization, the coupling relationship between curing age, strain rate and interfacial failure mode is further clarified. This work provides fundamental insights into the rate-dependent behaviors of polymer–cement heterogeneous interfaces, and offers theoretical guidance for the design of UPPC-based rapid repair systems.

## 1. Introduction

Thermoset polymer-based repair materials have attracted growing attention in infrastructure rehabilitation due to their fast curing, high early strength and excellent adhesion to inorganic substrates [1,2]. Among them, unsaturated polyester resin (UPR)-based polymer concrete (UPPC) [3,4] stands out as a cost-effective alternative to epoxy-based systems, featuring adjustable curing times, excellent chemical resistance, and good adhesion to mineral aggregates [5,6]. As a binary heterogeneous system consisting of a polymer repair layer and a cementitious substrate, the interfacial bonding zone directly governs the overall structural performance and service life of repair structures [7]. Extensive studies have confirmed that interfacial debonding is the dominant early failure mode of polymer-based repair systems, and interface performance has become the core bottleneck restricting the popularization of polymer repair materials [8].

Current characterization of the bond between polymer repair materials and ordinary Portland cement (OPC) substrates is predominantly conducted under quasi-static loading conditions [9,10]. Previous studies have confirmed that factors including substrate surface roughness, resin viscosity and curing age significantly affect static bond strength [11,12]. For UPR-modified cementitious systems, microscopic characterization has revealed that UPR incorporation can densify the interfacial transition zone (ITZ) and refine pore structures, providing a basis for understanding the static bonding mechanism [13]. Alameri et al. [12] further investigated the bond strength between polyester composites and concrete substrates and verified the enhancement effect of nanofillers on interfacial adhesion. However, most existing studies focus on static mechanical properties at full curing age, while the age-dependent evolution of interfacial bonding performance during the progressive polymerization of UPR remains poorly understood. In particular, the equivalent elastic modulus of the heterogeneous bonding interface is difficult to measure directly, which limits the quantitative characterization of interfacial mechanical development.

The dynamic mechanical behaviors of polymer concrete under impact loading have been extensively investigated for homogeneous bulk materials, but corresponding research on heterogeneous bonding interfaces remains scarce. In actual service scenarios, repair structures are inevitably subjected to dynamic flexural loads from vehicle impact, emergency braking and aircraft landing [14,15]. Ample evidence has demonstrated that homogeneous polymer concrete exhibits pronounced strain rate sensitivity, with dynamic strength and failure modes strongly dependent on loading rate [16]. For the UPPC system specifically, the authors have systematically characterized its dynamic mechanical properties from both experimental and numerical perspectives in previous work: dynamic compressive and splitting tensile responses at different curing ages were investigated via split Hopkinson pressure bar (SHPB) tests, verifying that the early-age strain-rate effect is more significant due to incomplete resin curing [17]. The dynamic mode I fracture behaviors of rubber-modified UPPC was further investigated, clarifying the coupling effect of loading rate and curing age on dynamic fracture performance [18]. In terms of numerical simulation, the dynamic constitutive model has been calibrated and applied to predict the low-velocity impact damage evolution of UPPC, providing a modeling basis for dynamic analysis of UPPC structures [19]. Nevertheless, all above studies focus on homogeneous UPPC bulk material, while the dynamic response of heterogeneous UPR-OPC bonding interfaces is far more complex and remains largely unexplored. The viscoelastic nature of partially cured UPR introduces unique rate-dependent mechanisms that differ from both brittle cementitious materials and fully cured polymer materials [20]. The dynamic increase factor (DIF) [21,22] is commonly used to describe the strain-rate effects for concrete materials. Similarly, to describe the strain-rate effect on bonding performance, it is also necessary to use DIF. It remains unclear how interfacial bond strength evolves over a wide strain-rate range, whether the DIF follows the conventional monotonic growth pattern observed in cement-based materials, and how the failure mode transitions with loading rate. 

This study systematically investigates the static and dynamic flexural bond performance of the UPPC-OPC interface across four curing ages (1 d, 3 d, 7 d and 28 d). Quasi-static three-point bending tests are first conducted to characterize the age-dependent development of bond strength and equivalent elastic modulus, with OPC-OPC bonded specimens set as the control group; high-strain-rate dynamic flexural tests are then performed using a 100 mm diameter SHPB system to analyze the strain-rate effect on interfacial bond strength and rate-dependent failure characteristics, and scanning electron microscopy (SEM) observation is further carried out to elucidate the underlying bonding mechanism. Specifically, this work systematically quantifies the evolution law of quasi-static flexural bond performance with curing age and verifies the significant early bonding advantage of UPPC over conventional cement-based repair materials through the control group, reveals an evolution pattern of the dynamic increase factor (DIF) of bond strength over a wide strain-rate range and clarifies the mechanical mechanism between viscoelastic weakening at low strain rates and rate hardening at high strain rates from the perspective of UPR polymerization kinetics, and establishes the coupling relationship among curing age, strain rate and interfacial failure mode via combined macroscopic mechanical tests and SEM microstructural characterization. The findings of this study are expected to provide supplementary experimental data and mechanistic insights into the rate-dependent behaviors of polymer–cement heterogeneous interfaces, and offer experimental guidance for the design of UPPC-based rapid pavement repair systems.

## 2. Mix Proportion and Experimental Arrangements

### 2.1. Raw Materials and Mix Proportion

The binder system of UPPC mainly consists of unsaturated polyester resin (UPR), curing agent methyl ethyl ketone peroxide (MEKP) and accelerator cobalt isooctanoate. The unsaturated polyester resin adopted is LY-191C orthophthalic UPR (Luye Brand, Zhaoqing, Guangdong, China), which features moderate viscosity and high reactivity. At 25 °C, the resin has a density of 1.05 g/cm^3^, a dynamic viscosity in the range of 400–600 mPa·s, an acid value of 18–24 mgKOH/g, and a solid content of 63–69%. The corresponding MEKP curing agent has a density of 1.10–1.12 g/cm^3^ at 25 °C with an active oxygen content of 9–10%, while the cobalt isooctanoate accelerator has a density of 1.0–1.10 g/cm^3^ at 25 °C and a cobalt mass fraction of 1%. In addition to the resin binder system, P.O 42.5R ordinary Portland cement (Shijing Brand, Guangzhou, Guangdong, China) is used as mineral filler. Crushed gravel with a particle size range of 4.75–9.5 mm and river sand with a particle size range of 0–4.75 mm is employed as coarse aggregate and fine aggregate respectively. Both coarse and fine aggregates are oven-dried to constant mass prior to mixing to avoid the adverse effect of moisture on the curing process of the resin. The detailed mix proportion of the UPPC mixture is specified in Table 1, where all aggregate masses are oven-dried.

### 2.2. Specimen Preparation

Unlike conventional cement concrete, UPPC was cast following a specific mixing procedure in this experiment. First, coarse aggregate, fine aggregate and cement were weighed and poured into a concrete mixer and then mixed for 60 s to achieve homogeneity. Separately, the unsaturated polyester resin was weighed into a container, followed by the addition of the pre-weighed MEKP curing agent. After 30 s of thorough stirring, the cobalt isooctanoate accelerator was added to the resin-curing agent mixture and stirred for another 30 s. Finally, the resin mixture was transferred into the concrete mixer and blended with the pre-mixed solid constituents for 2 min. Upon completion of mixing, the measured density of fresh concrete was 2300 kg/m^3^. In addition, the fresh resin concrete was cast into oiled molds and demolded after 12 h of curing at room temperature.

**Figure 1:**
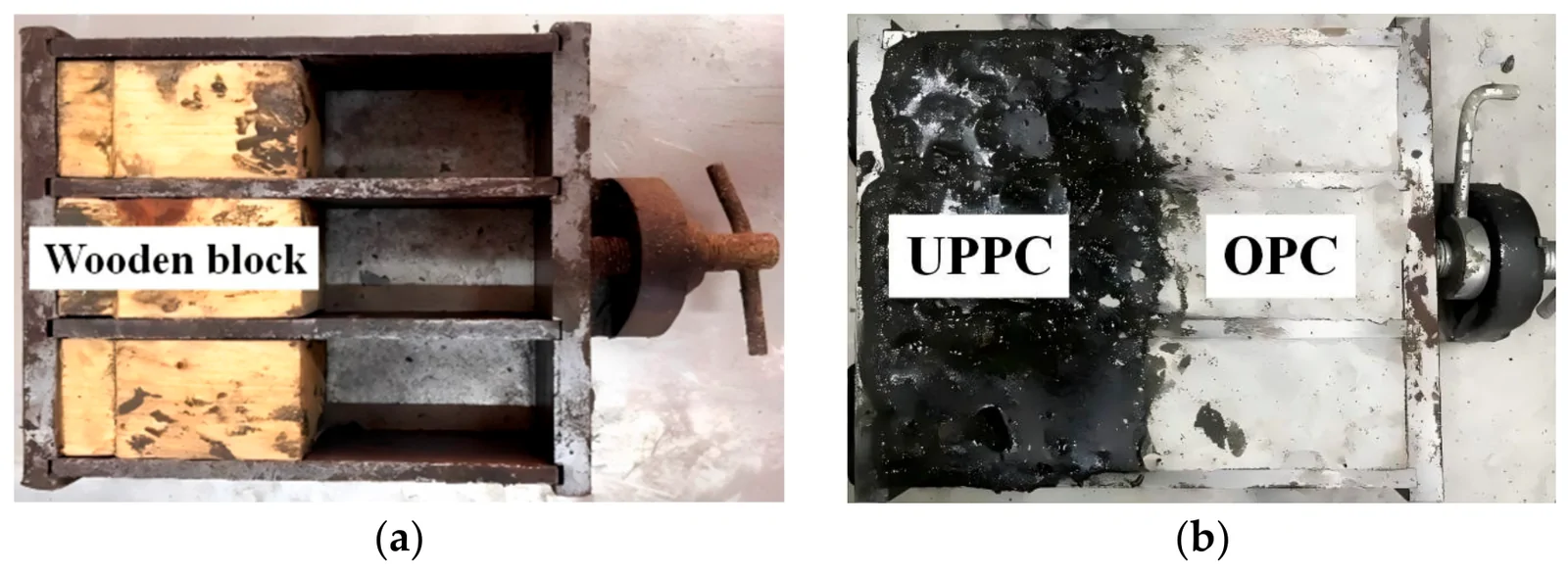
Photograph of the bonding specimen preparation. (**a**) The mold with wooden blocks; (**b**) casting of the bonding specimen.

To investigate the bonding performance between UPPC and existing concrete pavement, standard bonded specimens were fabricated in accordance with the Chinese standard JC/T 2381-2016 [23] to simulate the field repair condition. A photograph of the as-cast bonded specimen is shown in Figure 1a. To eliminate the influence of size effect on the experimental results, the specimen size for dynamic and static tests is consistent. The preparation procedure was as follows. A 40 mm × 40 mm × 160 mm flexural mold was used, and a prefabricated wooden block of 40 mm × 40 mm × 80 mm was placed inside the mold prior to casting, as illustrated in Figure 1b. The other half of the mold was then filled with OPC. The OPC substrate was prepared with P.O 42.5R cement, with a mix proportion of cement:water:sand:coarse aggregate = 1:0.52:1.53:2.97, and its 28 d cylindrical compressive strength was measured as 50.2 MPa. After casting and initial setting, the wooden block was removed, and the OPC substrate was cured for 28 days to simulate the aged pavement base. After 28 days of curing, a thin layer of pure UPR binder was applied to the bonding surface of the OPC substrate. After 1 h of penetration, UPPC was cast onto the treated OPC surface (Figure 1b), and the bonded specimens were demolded upon reaching the target curing ages. Four curing ages were selected for the bonding tests: 1 d, 3 d, 7 d and 28 d.

In addition, an OPC-OPC control group was prepared for the comparison with the UPPC-OPC bonded specimens. Both halves of the control specimens were fabricated using the same OPC mix proportion (cement:water:sand:coarse aggregate = 1:0.52:1.53:2.97). Specifically, one half of the OPC substrate was cast first and cured for 28 days to simulate the aged pavement base. Prior to casting the second half, a thin layer of pure cement paste was applied to the bonding surface of the pre-cured substrate. After 1 h of penetration, the fresh OPC mixture was cast onto the treated surface to form the integral bonded specimen. All OPC-OPC control specimens were cured under identical conditions as the UPPC-OPC specimens for the entire testing period.

### 2.3. Experimental Arrangements and Analytical Methods

#### 2.3.1. Material Characterization Tests

**Figure 2:**
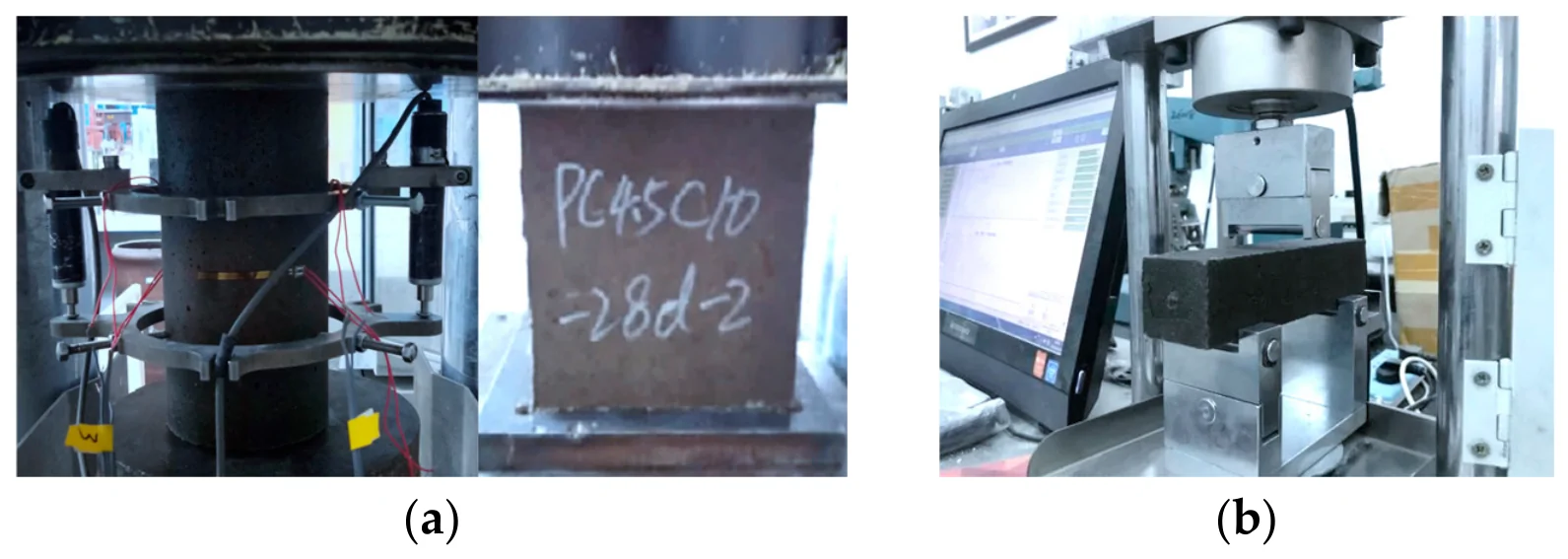
Photograph of the material characterization experiments. (**a**) Photograph of uniaxial and cubic compression tests; (**b**) photograph of a quasi-static flexural test for UPPC.

The fundamental mechanical properties of UPPC at four curing ages (1 d, 3 d, 7 d and 28 d), including cubic compressive strength and uniaxial compressive stress–strain behavior, were characterized using a Matest universal testing machine. Both cubic compressive tests and uniaxial compressive measurements were performed in accordance with the specifications of Chinese standard GB/T 50081 [24], and the test setup is shown in Figure 2a.

Quasi-static three-point flexural tests were conducted on a YAW-300C servo-controlled flexural–compression testing machine. All specimens were loaded at a constant stress rate of 0.10 MPa/s, corresponding to a nominal strain rate of approximately 1 × 10^−5^ s^−1^, in full compliance with the ASTM C293 standard [25]. For each curing age, the reported flexural strength was calculated as the average value of three valid replicate specimens according to Equation (1), where *P* is the maximum load; *L*_0_, *b*_0_ and *h*_0_ are the length, width and height of the specimen. The schematic of the test configuration is illustrated in Figure 2b.

(1) $$f_{s}=\frac{3PL_{0}}{2b_{0}h_{0}^{2}},$$

#### 2.3.2. Quasi-Static Interfacial Bond Tests

**Figure 3:**
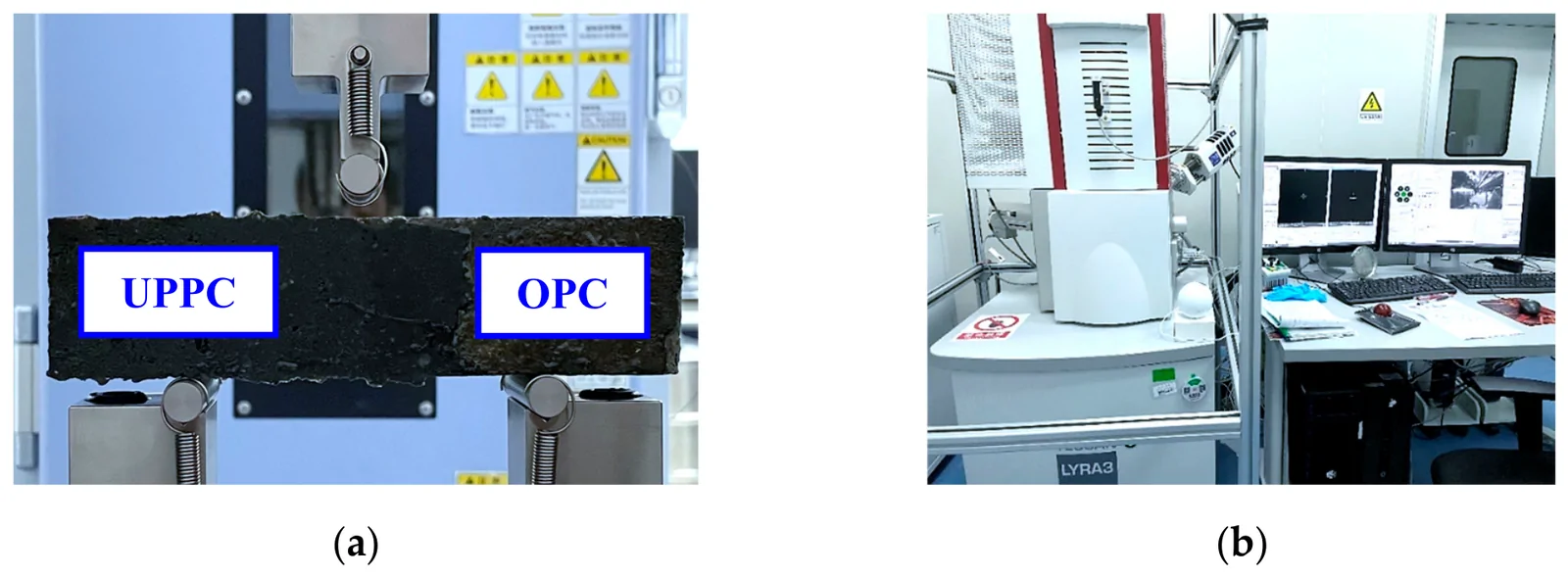
Photograph of the quasi-static interfacial bonding experiments. (**a**) Photograph of the flexural test on a bonding specimen; (**b**) photograph of the SEM machine.

Quasi-static three-point flexural bond tests on the repaired specimens were performed on a universal testing machine following the identical loading protocol (0.10 MPa/s), as depicted in Figure 3a. The interfacial bond strength (*f_rs_*) was also derived from Equation (1) to ensure calculation consistency. To further elucidate the bonding mechanism and failure characteristics at the microscale, the micromorphology of the UPPC matrix and the ITZ between UPPC and the OPC substrate were examined using a LYRA3 XMU focused ion beam field-emission scanning electron microscope (SEM), as shown in Figure 3b.

#### 2.3.3. Dynamic Interfacial Bonding Experiments

**Figure 4:**
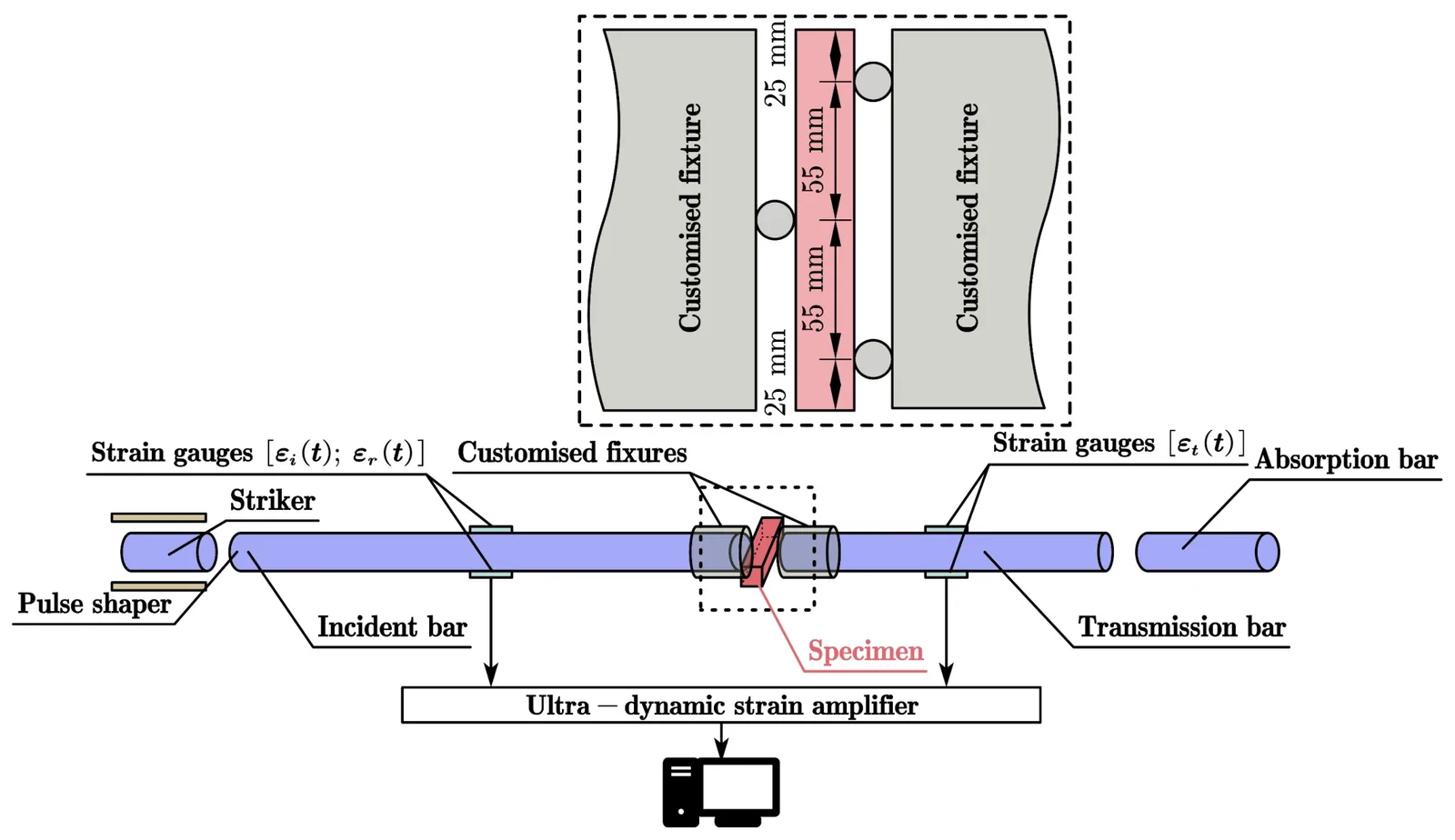
Diagram of the dynamic three-point flexural test.

Dynamic three-point flexural tests were performed on an SHPB (LWKJ-HPKS-Y100, Luoyang Liwei Technology Co., Ltd., Luoyang, Henan, China) with a diameter of 100 mm. By adjusting the driving gas pressure of the SHPB system, specimens were loaded at various impact velocities to characterize the mechanical performance of UPPC across different strain rates. The bars were made of 9260 alloy steel, and the physical characteristics of the bars of SHPB are summarized in Table 2. A pair of customized fixtures were adopted to realize the three-point bending configuration, with one fixture fixed at the end of the incident bar and the other at the end of the transmission bar, as shown in Figure 4. To capture the stress wave signals, BE120-3AA-P300 resistance strain gauges (manufactured by Zhonghang Electronic Measuring Instruments Co., Ltd., Taizhou, Jiangsu, China) were bonded onto the top and bottom surfaces of both the incident and transmission bars prior to testing. The axial distance between the two gauges on each bar was precisely controlled to exactly half of the bar circumference to eliminate the interference of bending waves on the measurement. Each strain gauge has a nominal resistance of 120 ± 0.2 Ω and a gauge factor of 2.22. The pulse waveforms collected by the strain gauges were recorded by a DH5960 ultra-dynamic strain amplifier (Jiangsu Donghua Testing Technology Co., Ltd., Taizhou, Jiangsu, China) with a sampling frequency set to 1 MHz. To achieve a nearly constant loading rate, dampen the high-frequency oscillations generated by the direct impact of the striker bar on the incident bar, and obtain a smooth incident pulse, a T2 pure copper disk with a thickness of 2 mm and a diameter of 20 mm was employed as the pulse shaper in all dynamic tests [26]. At least three replicate specimens with similar strain rates were tested for each strain rate condition. For each age group, to obtain the experimental data at three similar strain-rate levels, three impact velocities (approximately 1.5 m/s, 3.0 m/s and 4.5 m/s) were used to drive the striker to the incident bar. Three replicate specimens were prepared for each initial velocity to ensure the reliability of the test data.

The dynamic three-point flexural bond test is performed using the SHPB apparatus, which is fundamentally based on the theory of one-dimensional stress wave propagation [27], with elastic wave signals measured throughout the loading process. According to the classical momentum conservation equations and kinematic compatibility conditions, the load applied on the specimen *F*(*t*), the mid-span displacement *u*(*t*), and the velocity at the mid-span impact point *v*(*t*) can be determined as follows:

(2) $$F\left(t\right)=E_{b}A_{b}\left(\varepsilon_{i}\left(t\right)+\varepsilon_{r}\left(t\right)\right);$$

(3) $$v\left(t\right)=-C_{b}\left(\varepsilon_{i}\left(t\right)-\varepsilon_{r}\left(t\right)\right);$$

(4) $$u\left(t\right)=\int v\left(t\right)dt,$$

where *ε_i_*(*t*) and *ε_r_*(*t*) represent the incident wave and reflected wave signals collected by strain gauges mounted on the incident bar, respectively.

#### 2.3.4. Euler–Bernoulli Elastic Beam Model for the Infinitely Long Beam

In contrast to quasi-static loading conditions, dynamic loading acts on the specimen for an extremely short duration while delivering concentrated impact energy. The time required for a stress wave to propagate from the mid-span impact point to the free edge of the specimen, reflect off the free surface, and return to the mid-span loading position is defined as *T*_0_, Correspondingly, the duration of the bending test, that is, the time elapsed from the onset of loading to complete specimen fracture is denoted as *T_b_*. When *T_b_* exceeds *T*_0_, the stress wave is able to propagate fully to the beam supports before fracture occurs. In this case, the specimen approximately reaches a quasi-static equilibrium state, and the reaction force at each support equals half of the applied load.

Under high-strain-rate impact loading, however, the incident stress wave travels through the specimen thickness *h*_0_ and reaches the bottom surface. After that, the stress wave undergoes repeated diffraction and reflection within the specimen rather than propagating directly to the supports. As a typical brittle material, concrete undergoes an extremely rapid fracture process. As a result, the stress wave can only reflect a limited number of times inside the specimen before complete failure takes place. At the instant of fracture initiation, the stress wave has not yet reached the specimen support, leading to a zero-support reaction. In other words, beam failure occurs far earlier than the arrival of stress waves at the supports and specimen edges, so that the supports exert no reaction force during the fracture process. Physically, this means that no transmitted wave signal can be captured by the transmission bar in the SHPB test, i.e., *ε_t_*(*t*) ≡ 0 throughout the loading process [28].

**Figure 5:**
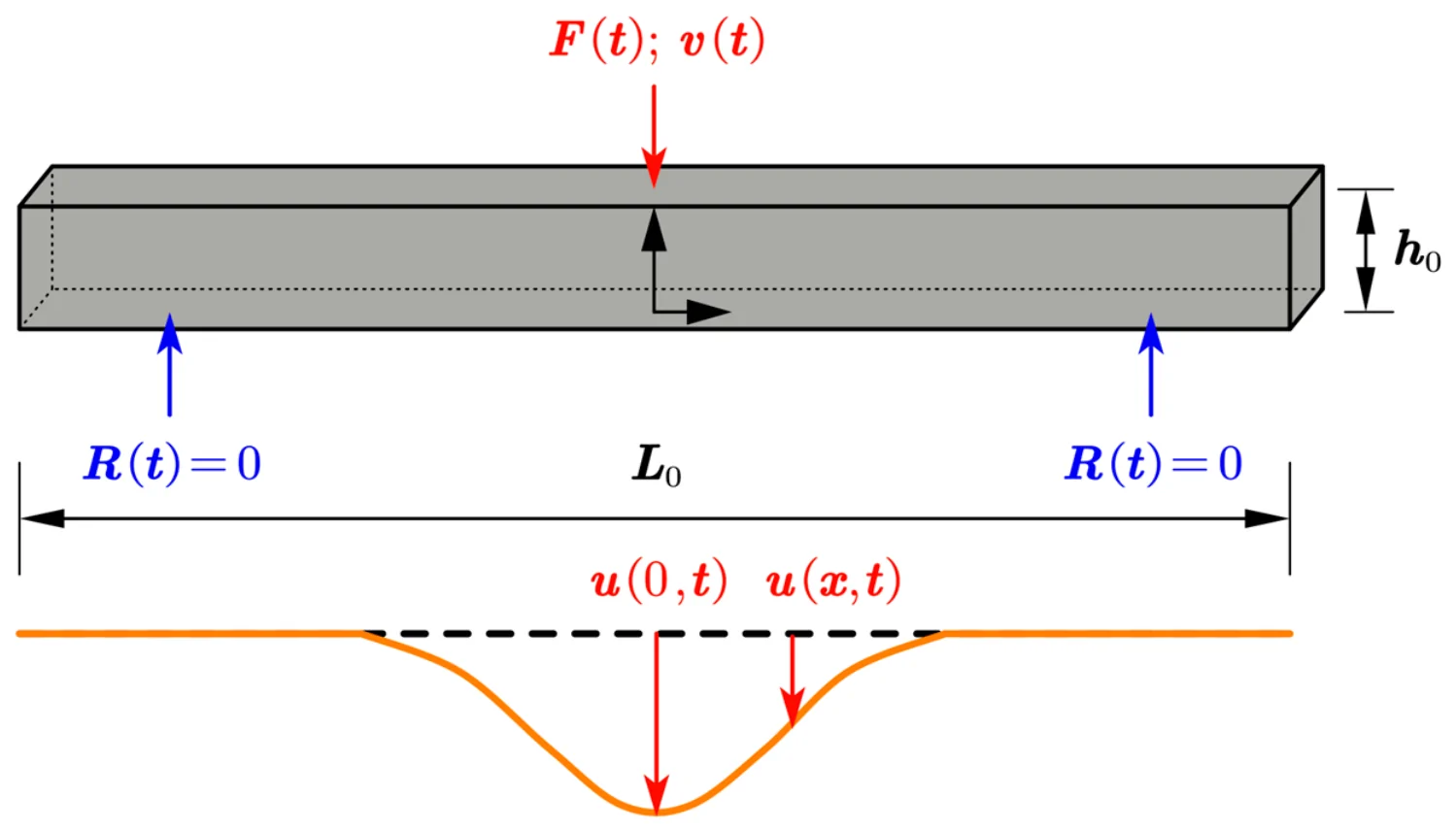
Schematic of the Euler–Bernoulli elastic beam model.

Under such conditions, the beam can be approximated as an infinitely long beam with a thickness-to-span ratio *h*_0_/*L*_0_ ≈ 0, which satisfies the fundamental assumptions of Euler–Bernoulli elastic beam theory [29]. The governing partial differential equation for the transverse vibration of the beam is written as:

(5) $$\frac{\partial^{4}u\left(x,t\right)}{\partial x^{4}}+4\alpha^{4}\frac{\partial^{2}u\left(x,t\right)}{\partial t^{2}}=0,$$

where $\alpha =\sqrt[{4}]{\rho_{0}S_{0}/4E_{0}I_{0}}$, *ρ*_0_ and *E*_0_ are the density and elastic modulus of the specimen, respectively; *S*_0_ is the cross-sectional area of the specimen; *I*_0_ is the moment of inertia of the beam cross-section. The schematic of the Euler–Bernoulli elastic beam model is illustrated in Figure 5, where the transverse displacement is zero at the infinite end of the beam (*x* = +∞). Delvare et al. [30] demonstrated that by applying the Laplace transform with respect to time, the transient dynamic response of the specimen can be expressed in terms of the dynamic force *F*(*t*) and velocity *v*(*t*):

(6) $$u\left(x,t\right)=\int_{0}^{t}G_{1}\left(t-\tau \right)\Omega_{1}\left(x,\tau \right)d\tau -\int_{0}^{t}\left[G_{1}\left(t-\tau \right)+G_{2}\left(t-\tau \right)\right]\Omega_{2}\left(x,\tau \right)d\tau ,$$

in which,

(7) $$\Omega_{1}\left(x,\tau \right)=\frac{1}{\sqrt{\pi t}}\cos\left(\frac{\alpha^{2}x^{2}}{2t}\right),\;\Omega_{2}\left(x,\tau \right)=\frac{1}{\sqrt{\pi t}}\sin\left(\frac{\alpha^{2}x^{2}}{2t}\right),$$

and

(8) $$G_{1}\left(t\right)=\int_{0}^{t}\frac{V\left(\tau \right)}{\sqrt{\pi \left(t-\tau \right)}}d\tau ,G_{2}\left(t\right)=\int_{0}^{t}\frac{F\left(\tau \right)}{4E_{0}I_{0}\alpha^{3}}d\tau ,$$

Fracture consistently initiates at the mid-span of the specimen (*x* = 0). Combined with the simply supported boundary conditions and the Euler–Bernoulli beam assumption, the relationship between the flexural stress at the specimen center and loading time can be expressed by Equation (9), where *v*(*t*) can be calculated by Equation (3). Extensive studies [31] on cementitious materials have confirmed that the elastic modulus of concrete does not exhibit significant variation within the tested strain-rate range. Accordingly, the elastic modulus of concrete is assumed to be constant, and the strain rate of the specimen under dynamic flexural loading can be derived according to Equation (10). The strain-rate effect on strength is commonly quantified by the dynamic increase factor (DIF) [32], defined as the ratio of dynamic bonding strength to the corresponding quasi-static bonding strength (*f_rd_*/*f_rs_*), where *f_rs_* and *f_rd_* denote the peak quasi-static and dynamic bond stress obtained from Equation (9).

In addition, it can be inferred from Equation (9) that the elastic modulus of the material is a prerequisite for calculating stress in dynamic fracture tests. For homogeneous UPPC specimens, the elastic modulus obtained from static tests can be adopted as an approximation for dynamic analysis. However, for the bonded specimen composed of an OPC substrate and a UPPC repair part, the elastic modulus is non-uniformly distributed along the longitudinal direction of the beam. Since the bonding interface between OPC and UPPC acts as the dominant weak plane of the entire bonded specimen, the equivalent elastic modulus of the bonded specimen is essentially governed by the mechanical properties of the interfacial zone. 

To date, few studies have systematically quantified the equivalent elastic modulus of the bi-material concrete bonded specimens, and the direct, accurate measurement of this property remains a technical challenge in the field. To address this limitation, this study adopts the equivalent elastic modulus of the UPPC-OPC bonded specimen, which is derived from the peak mid-span load and its corresponding deflection using the classical simply supported beam deflection formula from structural mechanics ($u_{max}=FL_{0}^{3}/48E_{0}I_{0}$). Specifically, the parameter *E*_0_ in Equation (9) denotes the equivalent elastic modulus of the UPPC-OPC bonded specimen.

(9) $$\sigma \left(t\right)=h_{0}\alpha^{2}E_{0}v\left(t\right)$$

(10) $$\dot{\varepsilon }\left(t\right)=h_{0}\alpha^{2}\frac{dv\left(t\right)}{dt}$$

## 3. Experimental Results and Discussion

### 3.1. Material Characterization

A series of quasi-static tests were carried out to systematically characterize the mechanical performance of UPPC at four curing ages (1 d, 3 d, 7 d and 28 d), covering uniaxial/cubic compressive behavior and three-point flexural behavior. The key test parameters are summarized in Table 3. In terms of compressive properties, UPPC presents a prominent ultra-early-strength development feature.

**Figure 6:**
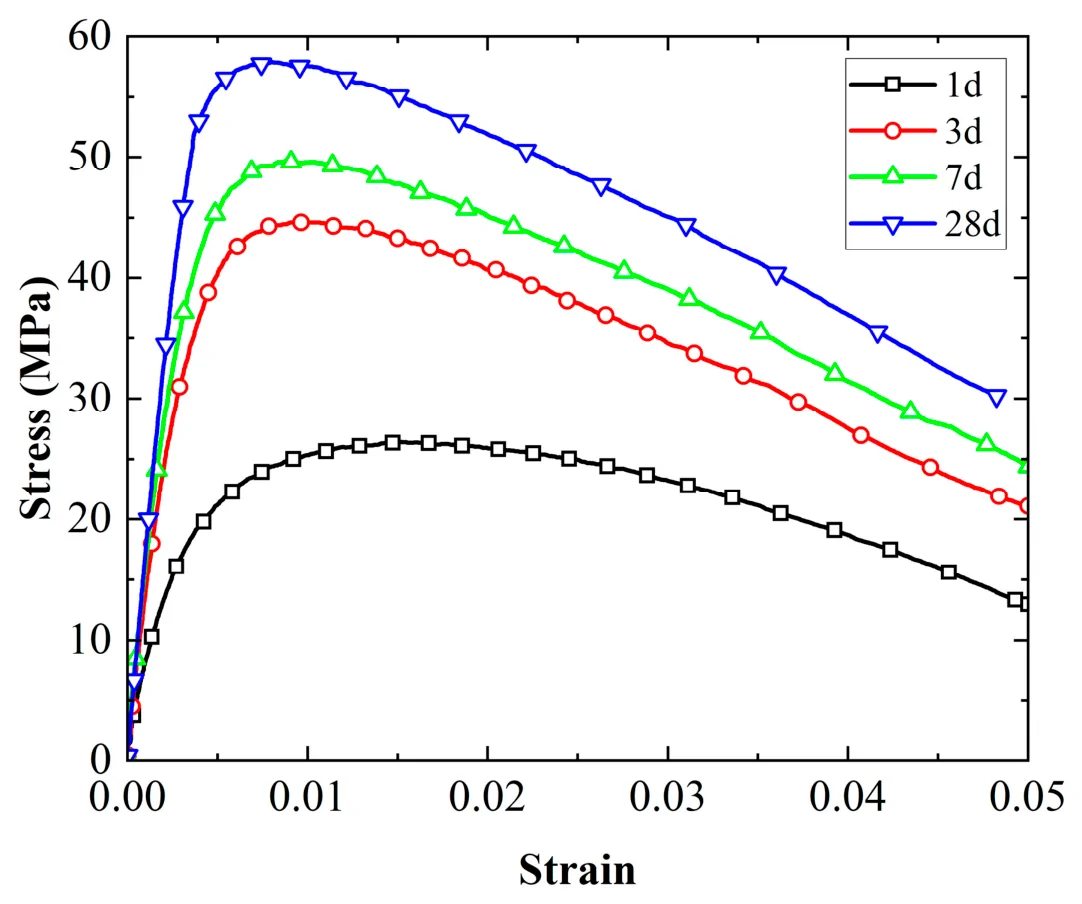
Uniaxial compressive stress–strain curves.

On the one hand, as shown in Table 3, the cubic compressive strength at 1 d reaches approximately 50% of its 28-day value, and both the uniaxial compressive strength and elastic modulus grow steadily with prolonged curing age. Compared with OPC at a similar compressive strength grade, UPPC has a considerably lower elastic modulus, which helps narrow the stiffness gap between the repair part and the existing base concrete and improves interfacial deformation compatibility [33]. Figure 6 illustrates the uniaxial compressive stress–strain curves of UPPC at different ages. All curves rise sharply in the initial loading stage, and after reaching the peak stress, they enter an extended softening stage with a gentle descending slope, corresponding to a fairly large ultimate compressive strain. In accordance with the specification in GB/T 50081, the ultimate compressive strain of UPPC exceeds 4%, which is far greater than that of OPC (approximately 0.33%). This result indicates that UPPC possesses excellent toughness and can absorb more deformation energy before fracture, which is conducive to resisting repeated vehicle loads in practical pavement applications.

On the other hand, the flexural strength of UPPC grows rapidly at early curing ages. The 1-day flexural strength reaches about 43% of the 28-day strength; at 3 days, the flexural strength rises to 70% of the 28-day value; by 7 days, the strength further increases to 18.4 MPa, reaching approximately 92% of the 28-day strength. Overall, the flexural strength of UPPC increases monotonically with curing age, but the growth rate declines progressively as curing proceeds, showing a typical trend of rapid strength gain in the early stage followed by gradual stabilization in the later stage.

In addition, to validate the feasibility of the calculation method of the equivalent elastic modulus of the bonding specimens, pre-tests on monolithic UPPC specimens were conducted prior to the quasi-static bond strength tests. The elastic modulus calculated via the deflection-based formula was compared with the value measured in strict accordance with the standard test specification. The comparison demonstrates that the results obtained by the two methods are in good agreement with only minor deviation, which confirms the reliability and applicability of the proposed method for equivalent elastic modulus determination of bonded specimens.

### 3.2. Quasi-Static Bonding Performance

#### 3.2.1. Failure Mode

**Figure 7:**
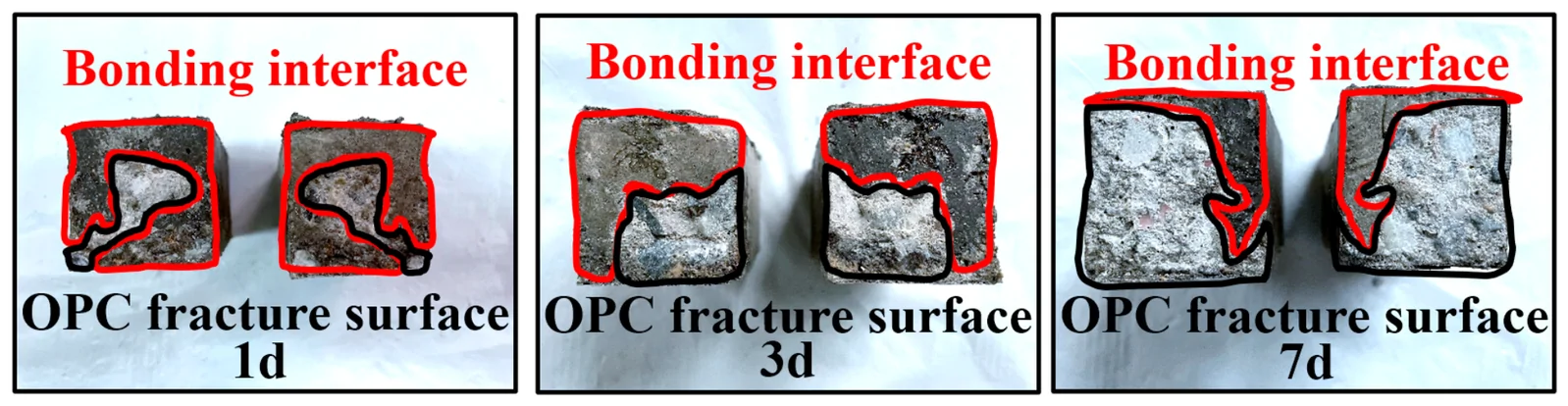
Fracture surface of bonding specimens under quasi-static loads.

Typical fracture morphologies of the UPPC-OPC bonded specimens after flexural failure at different curing ages are illustrated in Figure 7. The area of fracture OPC matrix on the failure surface expands continuously with prolonged curing age, with its proportion rising from approximately 28% at 1 d to 48% at 3 d and further to 72% at 7 d. This trend indicates that the overall flexural performance of the bonded specimen is governed not only by the flexural strength of UPPC itself, but also by the progressive development of interfacial bonding properties.

At the early age of 1 d, the adhesive bond between UPPC and the OPC substrate is relatively weak; fracture propagates predominantly along the bonding interface, with only a small fraction of OPC matrix torn off, corresponding to an interface-dominated failure mode. At 3 d, the interfacial bonding capacity improves gradually, and the increased proportion of OPC fracture area signals a transition from dominant interfacial debonding to a mixed mode involving partial matrix fracture. By 7 d, the fracture path extends deeply into the OPC substrate, and failure is dominated by matrix tearing. This phenomenon confirms that the interfacial bond strength has surpassed the tensile strength of the OPC surface mortar at this age, as fracture preferentially initiates and develops within the OPC substrate rather than along the interface, fully demonstrating the favorable repair effect of UPPC as a pavement repair material.

#### 3.2.2. Experimental Results

**Figure 8:**
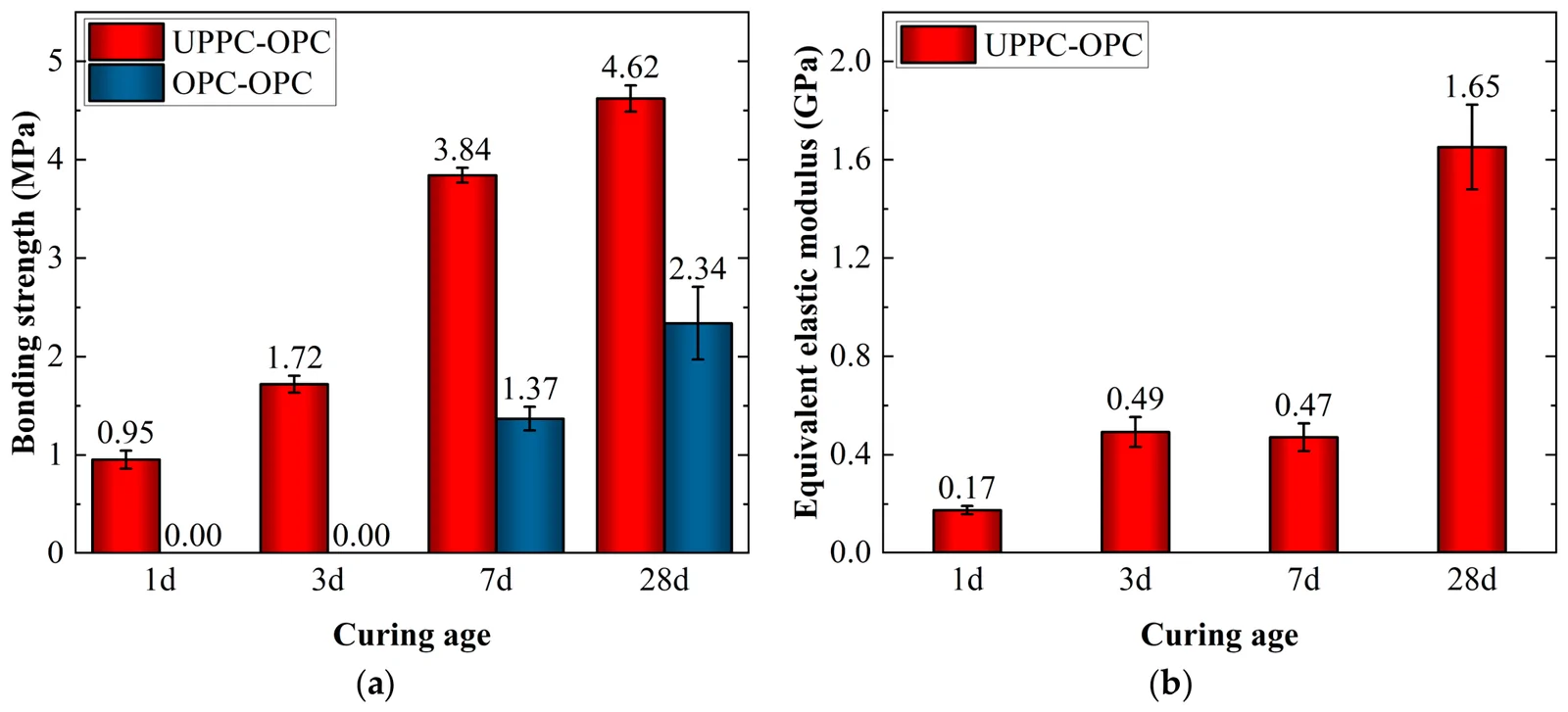
Relationship between bonding properties and curing age: (**a**) bonding strength; (**b**) equivalent elastic modulus.

The variation in interfacial bonding strength with curing age for both systems is shown in Figure 8a, where the UPPC-OPC system denotes that UPPC is considered as the repair material, and the OPC-OPC system reflects that OPC is considered as the repair material serving as the control group. For the OPC-OPC control specimens, interfacial fracture occurred directly upon demolding at curing ages of 1 d and 3 d, with virtually no measurable bonding strength. This result confirms that the early-age interfacial bonding capacity of conventional cement-based repair materials is severely inadequate, which fails to meet the strict requirements of rapid pavement repair engineering. For the UPPC-OPC bonded interface, the bonding strength exhibits a continuous upward trend with prolonged curing. At 1 d, the interfacial bonding strength reaches 0.95 MPa, endowing the repair structure with initial load-bearing capacity. The value increases to 1.72 MPa at 3 d, marking an approximately 81% rise compared with the 1-day strength. The growth rate accelerates further at the middle curing stage, as the bonding strength climbs to 3.84 MPa at 7 d. Eventually, the interfacial bonding strength of UPPC-OPC attains 4.62 MPa at 28 d. By contrast, the bonding strength of the OPC-OPC interface is only 1.37 MPa at 7 d and 2.34 MPa at 28 d, which is significantly lower than that of the UPPC-OPC system at all corresponding ages. Sprinkel and Ozyildirim [34] established a widely adopted grading criterion for the interfacial bond strength of concrete repair systems: bond strength below 0.7 MPa is rated as poor, 0.7–1.4 MPa as fair, 1.4–1.7 MPa as good, and above 2.1 MPa as very good. According to this criterion, the bond strength of the UPPC-OPC interface achieves the fair grade at 1 d, rises to the good grade at 3 d, and reaches the very good grade as early as 7 d. This rapid progression of bond performance further verifies the remarkable advantage of UPPC in interfacial adhesion when applied as a rapid pavement repair material.

The evolution of the equivalent elastic modulus of the UPPC-OPC bonding interface is presented in Figure 8b. In general, the equivalent elastic modulus increases progressively with curing age, consistent with the development trend of bonding strength. At 1 d, the equivalent elastic modulus stays at a relatively low level of 0.17 GPa, corresponding to the initial curing phase of the resin matrix. The values at 3 d and 7 d reach 0.49 GPa and 0.47 GPa, respectively, showing moderate growth and temporary stabilization in the early–middle curing stage. A substantial increment is observed at 28 d, with the equivalent elastic modulus rising to 1.65 GPa. Such evolution reflects that the curing reaction of the resin in the interfacial zone proceeds continuously over time, and the structural integrity and stiffness of the bonding interface are gradually enhanced as the curing age increases.

#### 3.2.3. Mesostructural Morphology of the Bonding Interface

**Figure 9:**
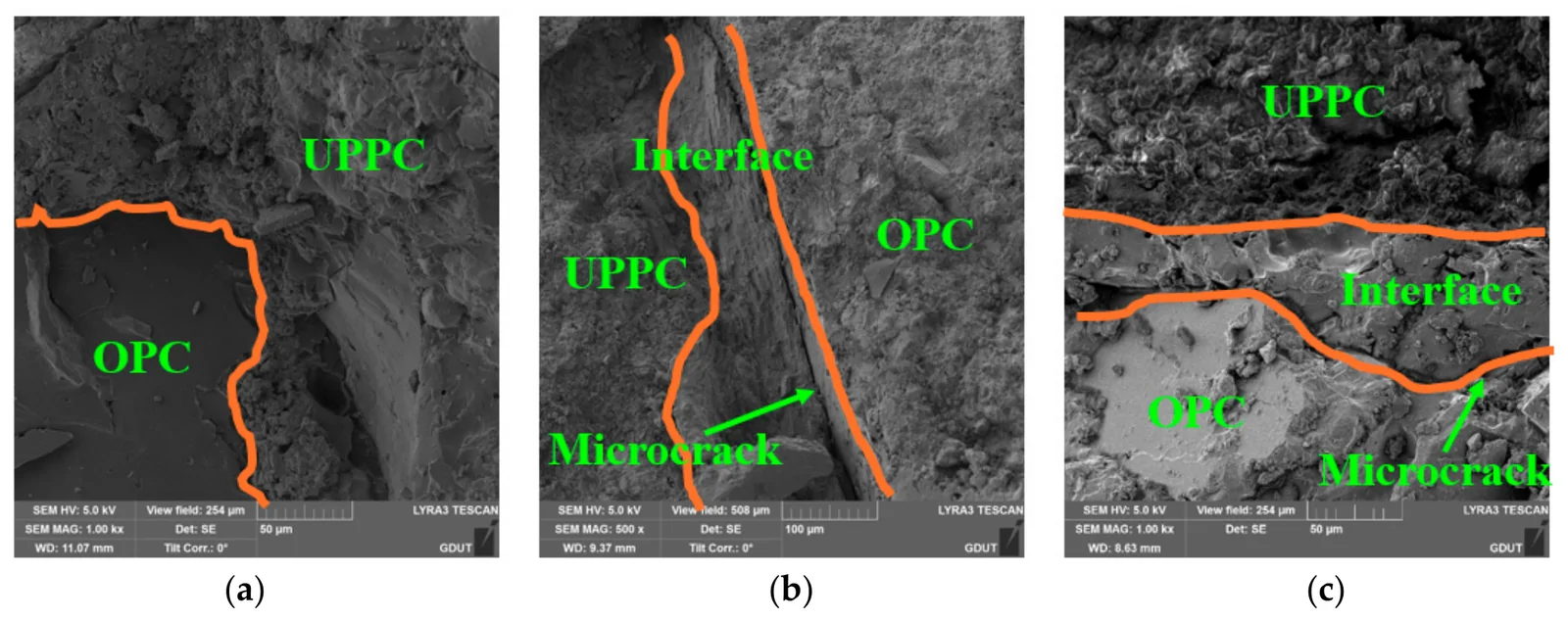
SEM images of the interfacial transition zone: (**a**) 3 d (×1000); (**b**) 7 d (×500); (**c**) 28 d (×1000).

The mesostructural morphology of the UPPC-OPC bonding interface at different curing ages is presented in Figure 9. Note that 1 d specimens were excluded from SEM characterization, as the interfacial UPR remained in the initial curing stage with a liquid-like viscous state and negligible crosslinking density, which would cause structural deformation under high-vacuum SEM observation. 

From the perspective of variable separation, three governing factors contribute to the age-dependent interfacial performance in different temporal stages: (1) Resin penetration is an early-age process completed before resin gelation. Low-viscosity UPR infiltrates into open pores and micro-defects on the rough OPC surface within hours after casting, forming a continuous gradient transition layer (ITZ). As shown in Figure 9a–c, the thickness of the ITZ does not increase notably from 3 d to 28 d, confirming that penetration no longer develops after initial setting. (2) UPR polymerization is the dominant factor driving continuous property growth throughout the curing period. For the 3 d cured specimen (Figure 9a), the UPR binder in the interfacial region is in the middle stage of free-radical polymerization, and the crosslinking degree and micromechanical properties of the ITZ are comparable to those of the bulk UPPC matrix. As curing proceeds, as shown in Figure 9b,c, resin polymerization within the bulk UPPC approaches completion at 7 days and 28 days, leading to a substantial improvement in matrix mechanical properties. (3) Moisture dilution from OPC pore water mainly retards the curing rate of interfacial resin. The curing process of the interfacial binder lags behind the bulk matrix due to the longer resin diffusion path into the OPC substrate and the dilution effect of pore moisture, resulting in a progressively widened property mismatch across the interface. This is indirectly verified by the fact that bulk UPPC reaches 92% of its 28 d flexural strength at 7 d, while the interfacial equivalent elastic modulus shows a sharp increment only after 7 d.

Additionally, distinct microcracks are observed along the interface–OPC boundary at both 7 days and 28 days, indicating that cracks preferentially initiate and propagate along this weak zone under loading. This mesoscopic feature provides microscale evidence supporting the interface-dominated failure mechanism revealed by macroscopic tests.

### 3.3. Dynamic Bonding Performance

#### 3.3.1. Failure Modes

**Figure 10:**
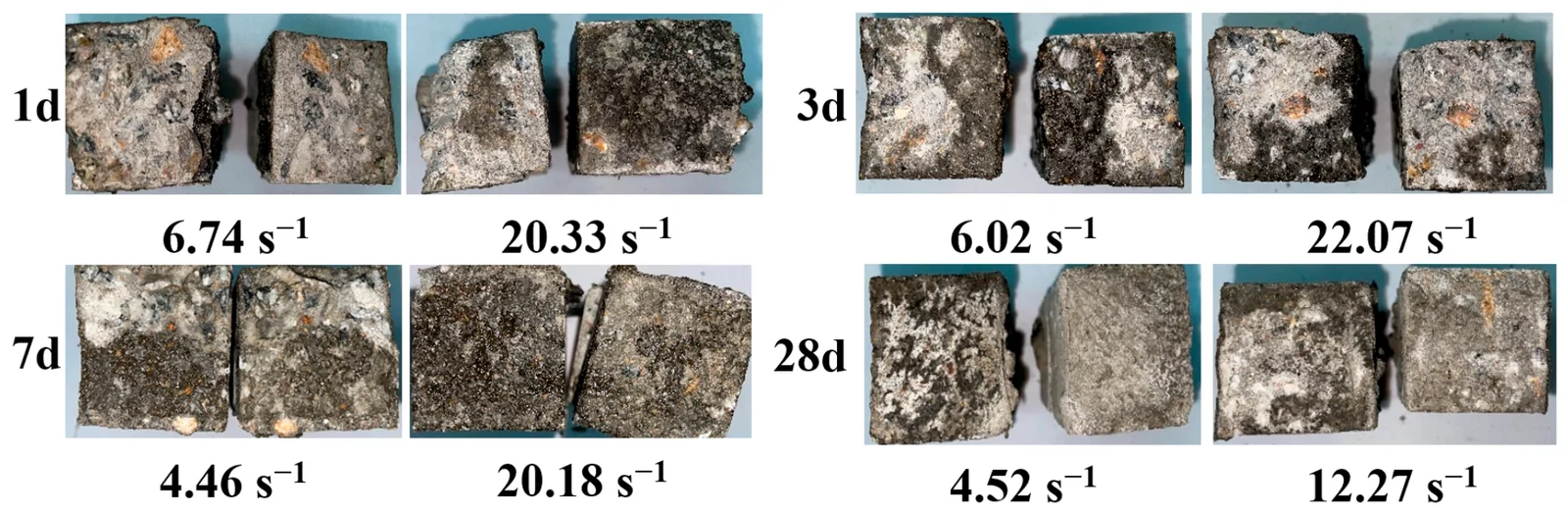
Fracture surface of bonding specimens under dynamic loads.

Typical fracture morphologies of UPPC-OPC bonded specimens at different curing ages and strain rates are presented in Figure 10. Typical fracture morphologies of UPPC-OPC bonded specimens at different curing ages and strain rates are presented in Figure 10. Compared with the quasi-static failure modes shown in Figure 7, dynamic loading introduces additional pronounced strain-rate dependence to the fracture characteristics.

The strain rate exerts a remarkable influence on the failure mode of the bonded specimens. Owing to the inherent heterogeneity of the bonding interface, cracks propagate preferentially along the weakest path under low-strain-rate loading. At relatively low impact velocities, cracks have sufficient time to deflect and extend into the superficial mortar layer of the OPC substrate, leaving a relatively large proportion of OPC mortar adhered to the fracture surface. By contrast, under high-strain-rate impact, cracks propagate at a much higher velocity with limited path deflection. Consequently, failure tends to take place directly along the bonding interface in a brittle debonding mode, with only a small amount of OPC mortar remaining on the fracture surface.

This discrepancy between low and high strain rates becomes more prominent with increasing curing age, indicating that the strain rate sensitivity of the interfacial bond is enhanced as UPR polymerization proceeds. With the improvement of interfacial strength, loading strain rate gradually becomes a key governing factor for the failure mechanism, shifting the dominant failure pattern from matrix-involved mixed fracture at low rates to pure interfacial debonding at high rates.

#### 3.3.2. Verification of the Euler–Bernoulli Infinite Beam Assumption

**Figure 11:**
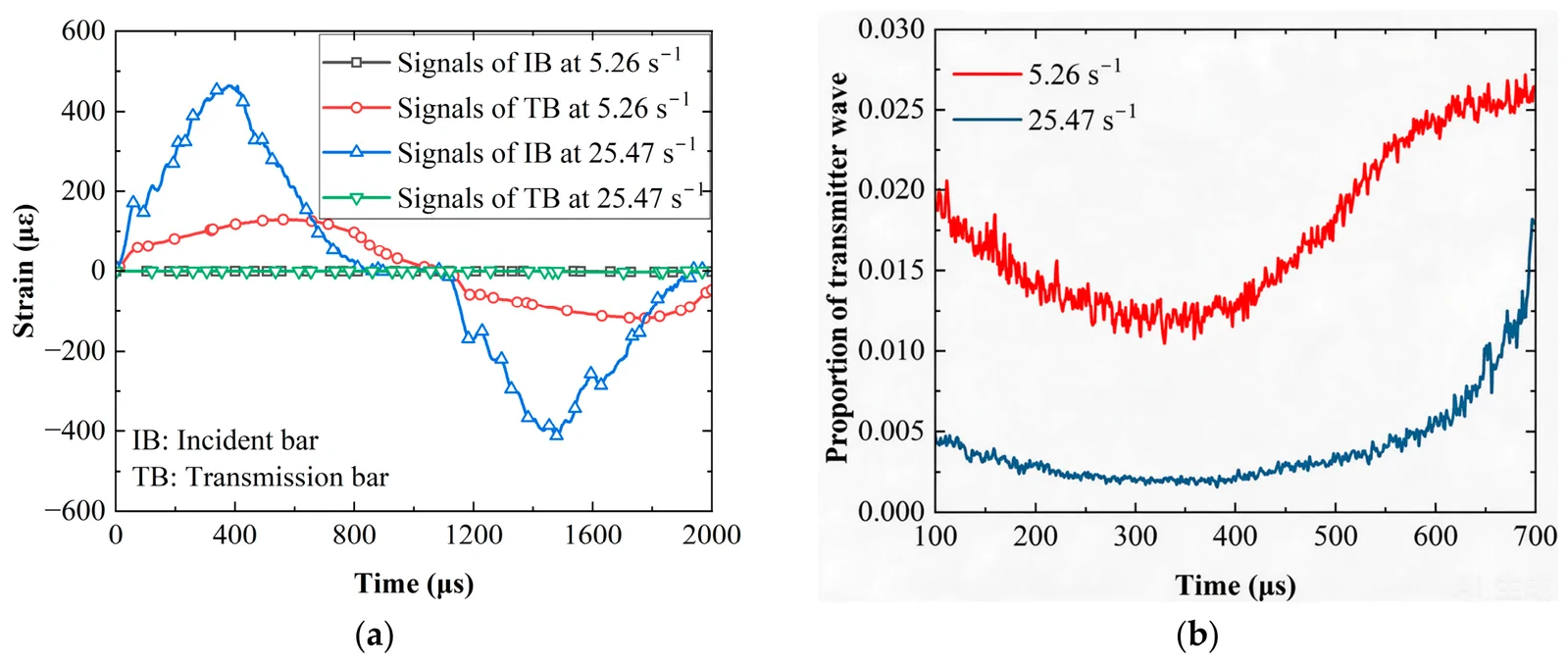
Verification of the Euler–Bernoulli infinite beam assumption: (**a**) strain–time histories of bonding specimens; (**b**) history of proportion of transmitted wave.

Figure 11a presents the raw strain–time histories collected by the ultra-dynamic strain amplifier under two typical strain rates, including signals measured from the incident bar and the transmission bar. At the initial stage of impact loading, the incident pulse is first captured by the strain gauge mounted on the incident bar. After propagating into the specimen, the stress wave undergoes multiple reflections and reverberations within the specimen and then travels backward along the incident bar. The returning wave is subsequently detected by the strain gauge on the incident bar, which forms the reflected wave signal. The onset of the reflected wave during signal processing is identified and extracted based on this propagation mechanism.

In addition, no valid strain signal is detected by the strain gauge on the transmission bar throughout the entire loading process. This phenomenon provides direct experimental support for the Euler–Bernoulli elastic beam assumption described above. In addition to the typical specimens in Figure 11a, no transmitted signal was detected in all valid specimens, confirming the applicability of the Euler–Bernoulli infinite beam assumption within the tested strain-rate range. As illustrated in Figure 11b, the amplitude ratio of the transmitted wave to the reflected wave (defined as (*ε*_t_(t)/*ε*_r_(t))) is consistently lower than 0.05 across the full loading duration. This result quantitatively confirms that the transmitted wave signal is negligible, which provides direct experimental support for the Euler–Bernoulli infinite beam assumption (i.e., the support reaction force remains zero during the fracture process). For brittle concrete specimens under three-point bending impact, the stress wave can only reflect a limited number of times inside the specimen before fracture initiates. Since specimen failure occurs prior to the arrival of the bending stress wave at the beam supports, the reaction force at the supports remains zero. Accordingly, the transmission bar in contact with the support bears no load during the whole test, which explains the complete absence of transmitted strain signals.

#### 3.3.3. Typical Examples of Calculating the Strain Rates, Stress and Mid-Span Displacement

**Figure 12:**
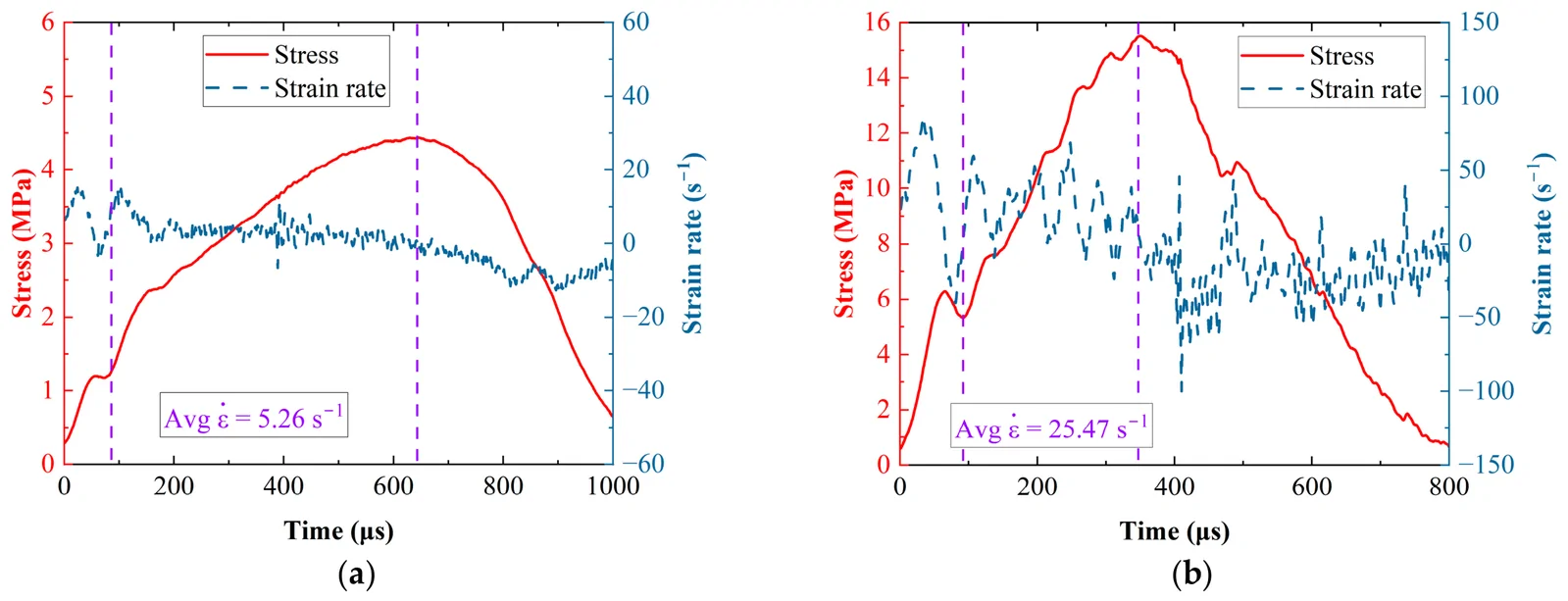
Typical stress and strain rate time-history curves of bonded specimen: (**a**) 5.26 s^−1^; (**b**) 25.47 s^−1^.

Typical stress and strain rate time-history curves of 28-day cured bonded specimens at two representative strain levels are presented in Figure 12 to illustrate the determination approach of the average dynamic strain rate. At the initial stage of impact loading, the instantaneous strain rate shows pronounced fluctuations, which can be attributed to the end-face contact compaction between the incident bar and the specimen as well as the inertial effect of the loading system. After the initial compaction phase, the strain rate gradually enters a relatively stable plateau with the steady propagation of stress waves inside the specimen, and the strain rate data within this stable interval are adopted to estimate the average strain rate of the specimen [35,36]. Accordingly, the average strain rates corresponding to the two typical cases in Figure 12a,b are determined as 5.26 s^−1^ and 25.47 s^−1^, respectively.

**Figure 13:**
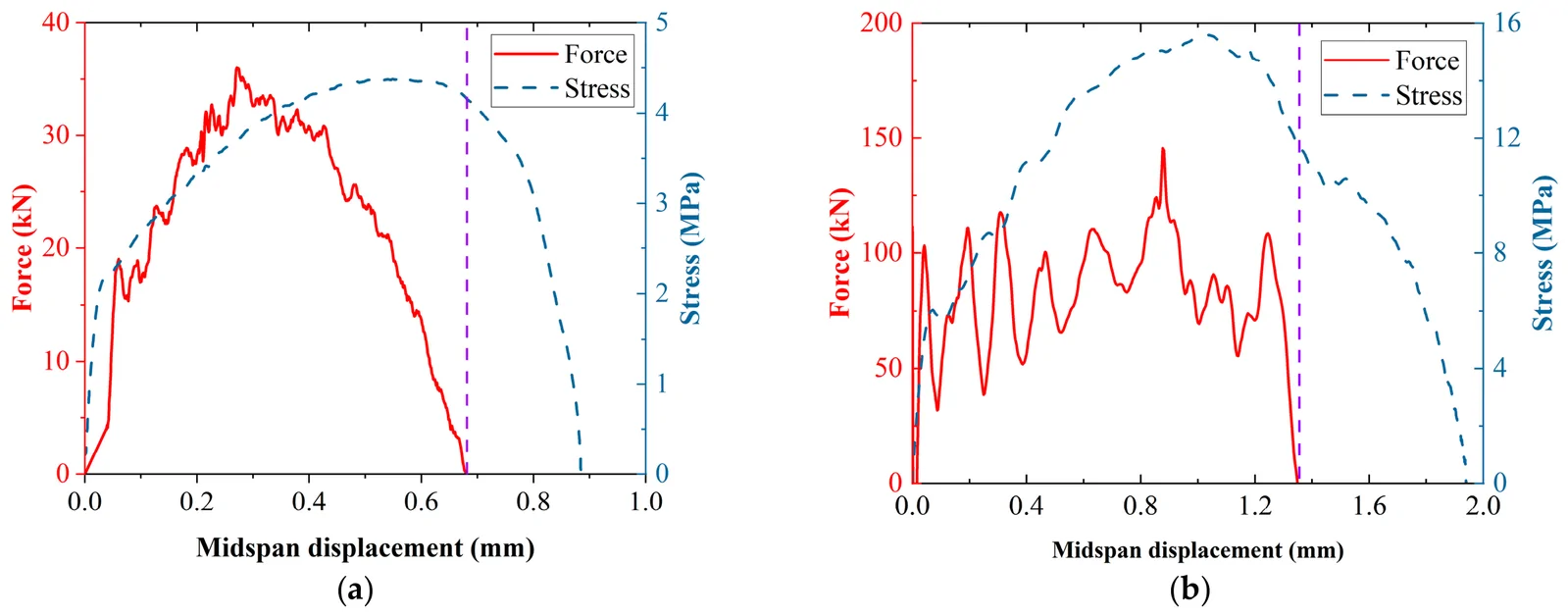
Relationship between stress, impact force, and mid-span displacement: (**a**) 5.26 s^−1^; (**b**) 25.47 s^−1^.

Figure 13 presents the relationships among impact force, flexural stress and mid-span displacement for the two corresponding bonded specimens in Figure 13, with the mid-span displacement derived from Equation (4). At a relatively low strain rate, the dominant crack propagates steadily along the weak bonding interface, giving rise to a smooth force–displacement curve with a single pronounced peak and slight fluctuations. By contrast, under high-strain-rate impact loading, the inherent heterogeneity of the bonding interface exerts a remarkable influence on the failure process. Owing to the uneven distribution of UPR thickness and variable bond quality along the interface, the interfacial stress state becomes highly non-uniform under rapid impact. The crack propagates alternately through weakly bonded and strongly bonded regions: in zones with strong interfacial adhesion where the bond strength exceeds the tensile strength of the OPC surface mortar, partial tearing of the OPC matrix occurs, whereas direct interfacial debonding dominates in weakly bonded zones. This alternating failure mechanism, coupled with the rate-dependent viscous behavior of the UPR, accounts for the pronounced oscillations and multiple peaks observed in the impact force curve. Given that concrete is a typical brittle material and the bonding interface acts as the primary weak plane, it is assumed in this study that the specimen fractures and separates from the incident bar immediately after the stress reaches its peak value. Accordingly, the mid-span displacement at the point where the impact force drops to zero is defined as the ultimate displacement of the specimen, which is determined as 0.68 mm and 1.31 mm for the specimens in Figure 13a and Figure 13b, respectively.

#### 3.3.4. Strain-Rate Effect of Bonding Strength and Ultimate Displacement

**Figure 14:**
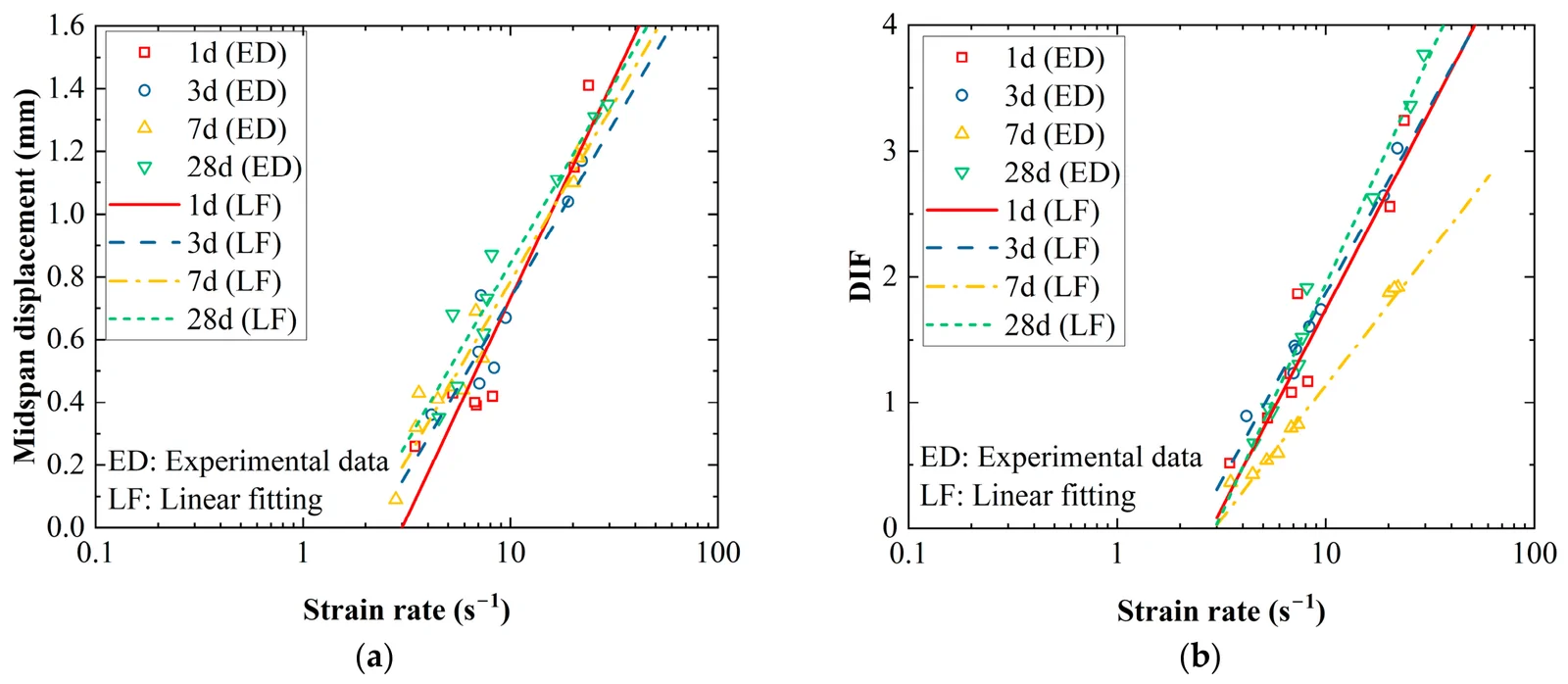
Strain-rate effect of (**a**) mid-span displacement and (**b**) DIF.

The strain-rate effect on the mechanical properties of concrete is generally analyzed by performing a linear fit between the logarithm of the strain rate and the mechanical properties [35,36,37]. Hence, Equations (11) and (12) describe the strain-rate effect of bonding strength (DIF) and ultimate displacement respectively, where *a*, *b*, *c*, *d* are the undetermined coefficients. Figure 14 and Table 4 present the strain-rate effects on the ultimate mid-span displacement and dynamic increase factor (DIF) of UPPC-OPC bonded specimens at four curing ages. As shown in Figure 14a, the ultimate mid-span displacement increases monotonically with rising strain rate for all curing ages, indicating that the bonding interface can sustain greater fracture deformation under higher impact loading, which aligns with the strength enhancement trend. The rate sensitivity of deformation capacity is generally comparable across the four curing ages, while 1-day specimens exhibit slightly stronger rate dependence than 3-day and 7-day specimens, which can be attributed to the highly viscous state of incompletely cured UPR at the early curing stage. For bond strength, Figure 14b demonstrates a pronounced evolution of DIF with strain rate. In the low-strain-rate range (below approximately 10 s^−1^) transitioning from the quasi-static regime, the DIF decreases to below 1.0, and this strength weakening phenomenon is most evident for 7-day cured specimens. With further increase in strain rate, the DIF rises progressively and eventually exceeds 1.0, exhibiting a distinct strain rate hardening effect. 

The underlying mechanism lies in the competition between viscoelastic interfacial adaptation at low loading rates and rate hardening plus inertial effects at high loading rates, which is jointly governed by UPR polymerization degree, resin penetration-induced mechanical interlocking, and pore moisture dilution, with each factor playing a distinct role: (1) UPR polymerization degree dominates the rate-dependent viscoelastic behavior. Under quasi-static conditions, the partially cured viscous UPR undergoes sufficient deformation to conform to micro-asperities and surface pores on the OPC substrate, maximizing the effective contact area and fully mobilizing mechanical interlocking and adhesive forces. Meanwhile, stress concentrations at interfacial micro-defects are effectively relaxed via viscoplastic resin flow, raising the crack initiation threshold. When the loading rate enters the low dynamic range, the resin lacks adequate time to achieve stress relaxation [38], leading to reduced effective bonding area and earlier crack initiation at defect tips, which accounts for the DIF values below 1.0. This weakening effect is most prominent at 7 days of curing because the partially polymerized resin retains a high fraction of viscoelastic phases, amplifying the discrepancy between creep-enhanced quasi-static strength and rate-limited dynamic strength. (2) Resin penetration provides the baseline mechanical interlocking force established at early ages. It determines the upper limit of static bond strength but does not dominate the strain rate sensitivity of the interface. (3) Moisture dilution weakens the micromechanical properties of the interfacial resin phase and prolongs the viscoelastic stage, which further amplifies the low-strain-rate weakening effect at early−middle curing ages.

As the strain rate exceeds approximately 10 s^−1^, strain rate hardening and inertial effects gradually prevail and outweigh the weakening mechanism. First, as a typical rate-sensitive polymer, the UPR exhibits remarkable increases in elastic modulus and tensile strength under rapid loading, intrinsically enhancing the adhesive resistance of the interfacial phase. Second, inertial confinement at high impact velocities restricts crack opening and propagation, demanding higher driving stress for fracture development. Third, the failure mode transitions with increasing strain rate: at low strain rates, cracks propagate steadily along the weakest interfacial path in a pure debonding mode controlled by the weakest interfacial regions; at high strain rates, fast-propagating cracks cannot deflect to follow weak zones and instead cut through strongly bonded regions and even tear the superficial mortar layer of the OPC substrate, incorporating additional matrix fracture resistance into the measured bond strength.

(11) $$u=a\;\log_{10}\;\dot{\varepsilon }+b,\;\dot{\varepsilon }\geq 3\;s^{-1}$$

(12) $$DIF=c\;\log_{10}\;\dot{\varepsilon }+d,\;\dot{\varepsilon }\geq 3\;s^{-1}$$

## 4. Conclusions

The static and dynamic flexural bond performance of the UPPC-OPC interface at four curing ages (1 d, 3 d, 7 d, 28 d) was systematically investigated via quasi-static three-point bending tests, 100 mm diameter SHPB dynamic flexural tests and microscopic characterization, with the following key conclusions drawn:

(1) UPPC exhibits excellent ultra-early strength and toughness. Its 1 d flexural strength reaches 43% of the 28 d value, and the ultimate compressive strain exceeds 4% (12 times higher than that of ordinary OPC). The relatively low elastic modulus of UPPC reduces the stiffness mismatch with OPC substrate, effectively improving interfacial deformation compatibility under traffic loads.
(2) UPPC shows significant advantages in early interfacial bonding over conventional cement-based repair materials. The 1 d bond strength reaches 0.95 MPa to meet the initial load-bearing requirement for rapid traffic opening, and the 28 d bond strength reaches 4.62 MPa, which is 97% higher than that of OPC-OPC control specimens.
(3) The UPPC-OPC interfacial bond strength exhibits distinct strain-rate dependence under impact loads. Within the applied strain-rate range of this study, the DIF is observed to transition from weakening (<1.0) to strengthening (>1.0) at around 10 s^−1^. This behavior is governed by the competition between viscoelastic stress relaxation of partially cured UPR at low strain rates and polymer rate hardening plus inertial confinement at high strain rates.
(4) The interfacial failure mode is jointly regulated by curing age and loading strain rate. For early-age specimens (1–7 d), the fracture surface presents viscous characteristics of incompletely cured UPR, while 28 d specimens show dry, fully cured fracture morphology. Under low strain rates, cracks have sufficient time to deflect and propagate into OPC surface mortar, presenting a mixed failure mode with high proportion of substrate tear; under high strain rates, cracks propagate rapidly along the interface, presenting brittle interfacial debonding. The strain rate sensitivity of interfacial bond increases with curing age, as fully crosslinked UPR exhibits more pronounced rate-dependent mechanical behaviors.

The findings of this study provide preliminary engineering references for heavy-traffic pavement repair applications: the UPPC system achieves a very good bond grade at 7 d, greatly shortening the required curing period compared with conventional cement-based repair materials; the strength weakening effect in the 1–10 s^−1^ strain-rate range should be considered in dynamic load design, with a corresponding correction coefficient recommended to prevent overestimation of interfacial bearing capacity; and the transition to matrix-dominated fracture can serve as an intuitive acceptance criterion for on-site bonding quality. Several limitations should be acknowledged: all validations are performed purely at the laboratory scale without full-scale field repair data; the analysis relies on a one-dimensional flexural idealization, and multiaxial stress states and fatigue loading conditions are not covered; and interfacial performance may be sensitive to substrate moisture content, surface roughness preparation and batch-to-batch variations in resin viscosity. Future work will calibrate these reference parameters via full-scale pavement tests and long-term field monitoring, and further conduct in-depth quantitative analysis of the bonding mechanism in the 1–10 s^−1^ range as well as the decoupling of governing factors, to develop a standardized design methodology for UPPC pavement repair.

## Figures and Tables

**Table 1:** Mix proportion of UPPC.

Resin (kg/m^3^)	Curing Agent (kg/m^3^)	Accelerator (kg/m^3^)	Cement (kg/m^3^)	Sand (kg/m^3^)	Gravels (kg/m^3^)
352.8	3.5	3.5	873.6	159.6	714.0

**Table 2:** Physical properties of bars.

Striker Length (mm)	Incident Bar Length (mm)	Transmission Bar Length (mm)	Young’s Modulus of Bars *E_b_* (GPa)	Density of Bars (kg/m^3^)	Elastic Wave Velocity (m/s)
1000	5500	3500	206	7710	5169

**Table 3:** Material properties of UPPC.

Curing Age (d)	Cubic Compressive Strength (MPa)	Uniaxial Compressive Strength (MPa)	Elastic Modulus (GPa)	Flexural Strength (MPa)
1	42.8	26.4	7.5	8.6
3	56.6	45.3	12.2	14.5
7	66.2	49.8	13.0	18.4
28	81.5	58.6	16.1	20.1

**Table 4:** Fitting results of strain-rate effect.

Curing Age (d)	Ultimate Displacement Fitting			DIF Fitting		
	*a*	*b*	R^2^	*c*	*d*	R^2^
1	3.164	−1.425	0.952	1.400	−0.668	0.903
3	2.980	−1.115	0.978	1.120	−0.388	0.888
7	2.125	−0.947	0.988	1.136	−0.350	0.970
28	3.652	−1.711	0.983	1.145	−0.301	0.934

Note: The linear regression is performed within the strain-rate range of 3–30 s^−1^, corresponding to Equations (11) and (12).

## Data Availability

The original contributions presented in this study are included in the article. Further inquiries can be directed to the corresponding author.
